# Fault Diagnosis of Bearings with Small Sample Size Using Improved Capsule Network and Siamese Neural Network

**DOI:** 10.3390/s25010092

**Published:** 2024-12-27

**Authors:** Jarula Yasenjiang, Yang Xiao, Chao He, Luhui Lv, Wenhao Wang

**Affiliations:** College of Intelligent Manufacturing and Industrial Modernization, Xinjiang University, Urumqi 830017, China; 107552204305@stu.xju.edu.cn (Y.X.); hcxd@stu.xju.edu.cn (C.H.); lvluhui_1998@stu.xju.edu.cn (L.L.); 107552304336@stu.xju.edu.cn (W.W.)

**Keywords:** small sample, capsule network, Siamese neural network, SKNet

## Abstract

This paper addresses the challenges of low accuracy and long transfer learning time in small-sample bearing fault diagnosis, which are often caused by limited samples, high noise levels, and poor feature extraction. We propose a method that combines an improved capsule network with a Siamese neural network. Multi-view data partitioning is used to enrich data diversity, and Markov transformation converts one-dimensional vibration signals into two-dimensional images, enhancing the visualization of signal features. The dynamic routing mechanism of the capsule network effectively captures and integrates key fault features, improving the model’s feature representation and robustness. The Siamese network shares weights to optimize feature matching, while SKNet dynamically adjusts feature fusion to enhance generalization performance. By integrating the Siamese neural network with SKNet, we improve transfer efficiency, reduce the number of parameters, and lighten the model to reduce complexity and shorten transfer time. Experimental results demonstrate that this method can accurately identify faults under conditions of limited samples and high noise, thereby improving diagnostic accuracy and reducing transfer time.

## 1. Introduction

With the rapid development of modern industrial technology, various types of machinery are continuously advancing towards larger scale and higher precision, making the operational stability and safety of equipment a critical issue in industrial production [1]. As a core component of rotating machinery, the operating condition of rolling bearings directly affects the performance and safety of the entire machine [2]. However, in practical applications, bearing faults are often difficult to detect in a timely manner. Additionally, factors such as downtime for equipment and the cost of data collection limit the availability of sample data for fault diagnosis [3]. Therefore, researching fault diagnosis methods under small sample conditions is of significant theoretical importance and practical value.

Traditional bearing fault diagnosis methods primarily rely on vibration signal analysis [4], extracting features from the signals to identify the operating condition of the bearing. However, these methods typically require a large amount of sample data and are not effective in handling complex signals. Moreover, traditional fault diagnosis methods depend on experienced professionals for analysis, which introduces subjectivity and reduces efficiency. Therefore, exploring a new method that can automatically and accurately diagnose bearing faults, even under small sample conditions, has become a current research hotspot.

With the rapid development of big data and artificial intelligence technologies, deep learning and other machine learning algorithms have been widely applied in fault diagnosis [5]. For example, transformer [6], ResNet [7], long short-term memory networks [8], and particularly convolutional neural networks (CNNs) [9] have demonstrated significant potential in bearing fault diagnosis due to their powerful feature extraction and classification capabilities. Lei et al. [10] addressed the issue of low accuracy in bearing fault diagnosis when using traditional methods in complex and variable real-world conditions with limited datasets. They proposed the MTF-CNN model for rolling bearing fault diagnosis. This model uses Markov Transform Field (MTF) encoding to convert raw one-dimensional vibration signals into two-dimensional feature images with temporal correlations. These feature images are then input into a CNN for automatic feature extraction and fault diagnosis, ultimately achieving the classification of different fault types. Guan et al. [11] tackled the problem of long training times and low efficiency in traditional rolling bearing fault diagnosis methods by proposing a fault diagnosis approach based on CNNs and Broad Learning Systems (BLS). This method achieves end-to-end fast and accurate pattern recognition using a wide convolutional learning system that combines CNN for feature extraction and BLS for classification. The output is fed into a residual learning system with increased BLS layers to optimize the model output and identify fault patterns.

However, deep learning models typically require large amounts of training data to achieve good performance, which limits their application in small sample scenarios. How to effectively utilize deep learning models for bearing fault diagnosis under small sample conditions has become a hot topic. Wu et al. [12] summarized suitable transfer learning methods for different models under various conditions, overcoming the limitations of traditional deep learning methods that require large labeled datasets, have limited applicability, and lack generalizability. Zhao et al. [13] addressed the issues of low accuracy and poor generalization in traditional deep learning models under small sample conditions by proposing a bearing fault diagnosis method based on Improved Siamese Neural Networks (ISNN). This method uses metric learning to determine sample similarity, has a simple structure, and is easy to train with small samples. It consists of three modules: feature extraction using LSTM and CNN to extract spatiotemporal features, relationship measurement using adaptive network metrics and global average pooling to reduce parameters, and fault classification using a direct sample recognition network without the need for complex comparisons.

To better extract data features and improve the generalization and robustness of the model, this paper adopts an improved capsule network. Capsule Networks, compared to fully connected layers, not only inherit the local connectivity characteristics of CNNs but also enhance feature representation through the introduction of capsule units. This design significantly reduces the number of parameters in the model, providing an inherent regularization mechanism that helps prevent overfitting. Similar to CNNs, capsule networks exhibit strong robustness to affine transformations of image data, allowing for the full utilization of various affine transformations to enhance the model’s generalization capability and further reduce the risk of overfitting. Wang et al. [14] addressed the issues of limited fault samples and low pooling efficiency in CNNs by proposing a twin small-sample learning model based on capsule networks. This model classifies features by learning differences between sample pairs. Yuan et al. [15] addressed the issue of complex working conditions leading to compound faults in rolling bearings, which are difficult to identify using traditional methods, by proposing an intelligent diagnosis method based on capsule networks and multi-label classification. The method inputs vibration signals, extracts features through convolution and squeeze–excitation modules, converts features into vectors using primary capsule layers, and routes them to higher-level capsule layers using self-attention. Finally, a multi-label classifier provides the diagnosis results.

Based on the above analysis, this paper proposes a small-sample rolling bearing fault diagnosis method that combines capsule networks and Siamese neural networks. The proposed method mainly consists of three components: the data preprocessing module, the feature extraction module, and the Siamese capsule network module.

In the data preprocessing module, a multi-view data segmentation strategy is employed to split the data using different sliding windows and transform it into two-dimensional images using Markov transformation. This process generates feature vectors with different time scales and densities, and the Markov transformation converts these feature vectors into two-dimensional images. This not only retains important information from the original signals but also enhances the readability and interpretability of the data, contributing to improved model accuracy and generalization.

In the feature extraction module, an extended three-channel SKNet is used. Input images are processed through channels with more suitable convolutional kernels, which helps in feature extraction. This approach reduces the number of model parameters, lightens the diagnostic model, improves diagnostic efficiency, and reduces diagnostic time.

In the Siamese capsule network module, the source domain model is pre-trained with certain parameters frozen. The capsule network branch structure and weight parameters from the source domain fault diagnosis model are transferred to the target domain diagnosis model. The remaining network parameters are then fine-tuned and updated to enhance the performance of the Siamese capsule network in the target domain.

Finally, experimental results demonstrate that the proposed method outperforms other methods in terms of performance.

## 2. Related Work

### 2.1. Few-Shot Learning

In industrial environments, fault events are relatively rare and costly to collect, which limits the effectiveness of traditional supervised learning methods [16]. Therefore, this paper focuses on fault diagnosis under small-sample conditions, employing transfer learning as the core strategy to overcome the limitations of insufficient data.

Transfer learning [17] is an effective machine learning method whose core idea is to leverage knowledge learned from related domains or tasks to assist in solving problems with insufficient samples in the target domain. In this study, we designed a fault diagnosis framework based on transfer learning. We selected a source domain task that is similar to the target fault diagnosis task but has a larger dataset for pre-training to extract generalizable feature representations and build strong prior knowledge. Subsequently, this knowledge is transferred to the target domain, where fine-tuning or adaptive learning is applied to enable the model to quickly adapt to the limited samples in the target domain and effectively perform fault diagnosis.

The advantage of this method lies in its ability to significantly reduce the demand for data in the target domain while maintaining high diagnostic accuracy and accelerating the model’s learning speed. By leveraging few-shot learning and transfer learning techniques, the method can be trained on one device and applied to others, thereby maximizing its applicability and reducing reliance on large-scale labeled data. The model simultaneously learns diagnostic tasks for multiple fault types, further enhancing its generalization capability. This approach enables the model to diagnose not only bearing faults but also extend to fault diagnosis in other mechanical equipment.

### 2.2. Capsule Network

**Figure 1:**
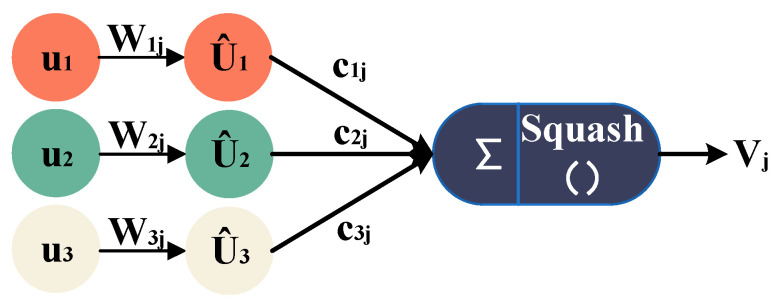
Schematic diagram of a capsule unit.

Capsule Network [18] is an innovative architecture in the field of deep learning designed to address the issue of losing spatial positional relationships in image recognition by traditional Convolutional Neural Networks (CNNs). As shown in Figure 1, the core of the Capsule Network lies in its basic unit—the capsule. Each capsule not only contains scalar values but also encapsulates feature vectors, which can represent various attributes of entities, such as orientation, angle, and length. This design preserves the spatial hierarchical relationships between entities.

**Figure 2:**
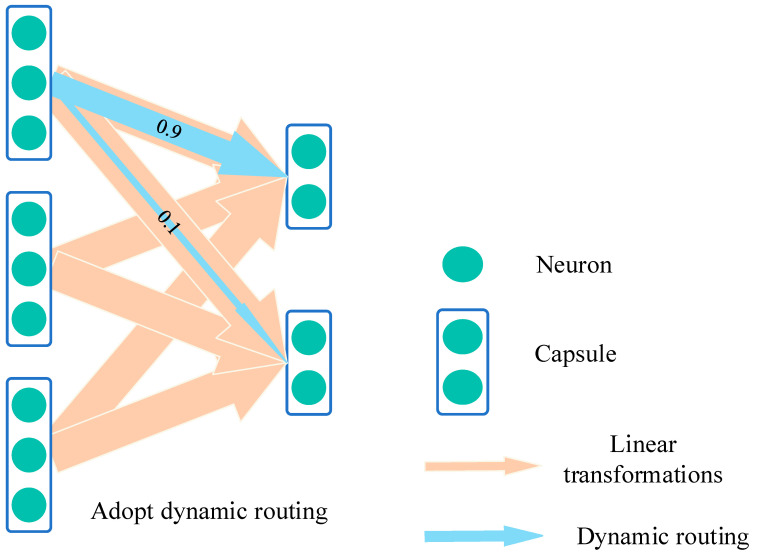
Schematic diagram of dynamic routing.

Unlike the pooling layers in CNNs, capsule networks use the dynamic routing mechanism [19], as shown in Figure 2, to transmit information. During the dynamic routing process, lower-level capsules dynamically adjust the paths of their output vectors based on the match between their prediction vectors and those of higher-level capsules. This mechanism enables capsule networks to more accurately identify objects and their attributes in images, enhancing the network’s ability to model spatial hierarchies and relationships and allowing it to capture deeper features. This capability is crucial for handling uncertainty and variability in samples. The process considers not only the magnitude of feature vectors but also the spatial consistency between features, permitting adaptive propagation of features across network layers. Each capsule node transmits not only its activation value but also corresponding “weight” information, indicating its “confidence” in specific features. This mechanism effectively propagates the inherent uncertainty of data from lower to higher layers, thereby improving the robustness of the entire network for small-sample data and enabling more accurate recognition and understanding of complex structures in images. The spatial hierarchical representation capability of capsule networks aids in pinpointing the exact location of faults and their relationships with other components, particularly evident when dealing with highly overlapping or complexly posed objects.

### 2.3. Siamese Network

**Figure 3:**
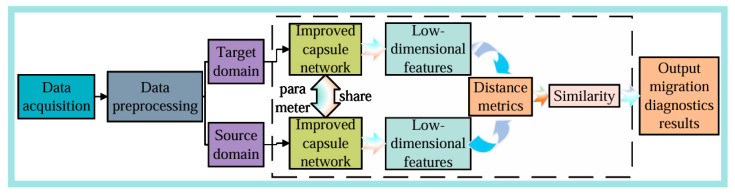
Schematic diagram of a Siamese capsule neural network.

Small-sample learning models primarily include prototypical networks [20], relation networks [21], matching networks [22], and Siamese networks [23]. Among these, prototypical networks provide support sets and query sets, transforming the classification problem into a nearest neighbor problem in the embedding space. Relation networks construct a neural network to calculate the distance between two samples to analyze the degree of match. Matching networks use two different embedding functions for the support set and query set, and they compute a weighted sum of the predictions for the support set samples and query set samples, which serves as the output of the classifier. Siamese networks build a parallel neural network with shared weights. As shown in Figure 3, this paper employs a Siamese capsule network for diagnosing bearing faults by determining the type of test samples based on their similarity to labeled samples. Through learning from paired samples, this approach reduces uncertainty caused by individual sample differences. Specifically, the Siamese network focuses on comparing the similarity between paired samples, allowing the network to concentrate on the relationship between two samples rather than the absolute values of single samples. This enhances the network’s adaptability to data variability and uncertainty, thereby achieving the goal of classifying samples.

#### Introduction to Distance Metric Functions

To measure the similarity between two feature vectors, the Euclidean Distance [24] is adopted as the metric function. Euclidean Distance is a simple yet effective metric that directly reflects the spatial distance between two vectors. In the Siamese Capsule Network, the model parameters are optimized by minimizing the Euclidean Distance between positive sample pairs and maximizing the Euclidean Distance between negative sample pairs, thereby enhancing the model’s ability to distinguish between different fault types. The following Table 1 outlines the steps and explanations for calculating Euclidean distance.

## 3. Proposed Method

### 3.1. Multi-View Joint Optimization for Feature Extraction

Subtle faults refer to those that are difficult to detect in their early stages and have a minor impact on system performance but may gradually deteriorate over time. These faults can be caused by factors such as material aging, micro-cracking, slight wear, or inadequate lubrication, manifesting as subtle vibrations, noise, temperature changes, or performance degradation. Traditional one-dimensional signal analysis methods often fail to promptly capture these minute changes, leading to delayed fault diagnosis. However, if not addressed in a timely manner, subtle faults can accumulate and lead to more severe structural failures. Therefore, early identification of subtle faults is of critical importance.

Compound faults arise from the simultaneous or sequential action of multiple fault sources or modes, posing significant diagnostic challenges. In structural systems, compound faults may present as intertwined mechanisms of corrosion, fatigue, and wear. The interaction between these fault modes complicates the expression of fault characteristics. Additionally, environmental factors such as temperature, humidity, and corrosive conditions significantly influence the progression of compound faults, further increasing the complexity of fault diagnosis.

In practical applications, structural systems are often subjected to various loads and environmental influences, including static loads, dynamic loads, temperature variations, humidity fluctuations, and corrosive environments. These factors can alter the stress distribution and deformation patterns of structures, thereby exacerbating the development of subtle or compound faults. Consequently, fault diagnosis in such complex environments requires more precise and sensitive monitoring technologies.

In this context, the Markov transform method effectively captures the temporal correlation and minute fluctuations of signals by converting one-dimensional signals into two-dimensional images. Unlike traditional methods, Markov transform converts subtle changes in amplitude and frequency into pixel differences in images, making these changes visually clearer and more distinguishable. This image-based processing not only enhances the visibility of subtle faults but also provides richer information for neural network models, thus improving their sensitivity to subtle faults.

For compound faults, Markov transform integrates multiple fault features into a single two-dimensional image, enabling neural network models to better understand the overall fault state. Given that compound faults involve multiple sources and complex interactions, the two-dimensional image format helps models more accurately capture these intricate fault patterns. Compared to one-dimensional signals, two-dimensional feature images contain more comprehensive fault information, effectively distinguishing different fault modes and enhancing the diagnostic capability of the model.

Markov transformation effectively preserves the local dynamic changes in vibration signals through the transition probability matrix, especially capturing short-term fault evolution characteristics when signal variations are minimal. The transition probability matrix encodes state transitions of the signal, revealing patterns of change between different states, making it particularly suitable for early fault diagnosis and detection. Although this method reduces computational complexity by simplifying signal features, it retains sufficient dynamic information to assist models in making accurate judgments during fault diagnosis. To further enhance the capture of temporal features, a sliding window approach with varying window sizes and strides is employed. These adjustments improve the adaptability of Markov transformation to dynamic fault evolution and contribute to extracting richer feature representations.

(1) Window Size 1024, Stride 40: This segmentation method generates images that are relatively dense along the time axis and have a moderate time span. Each image contains 1024 consecutive data points, with an overlap of 98.4% (i.e., (1024−40)/1024) between adjacent images, helping the model capture subtle changes in the signal;
(2) Window Size 1024, Stride 80: Compared to the first method, this generates images that are less dense along the time axis but contain more temporal information. The overlap between adjacent images decreases to 92.2%, aiding the model in understanding the overall trend of the signal from a broader perspective;
(3) Window Size 2048, Stride 40: By increasing the window size to 2048, this method generates images with a larger time span. Maintaining a stride of 40 ensures a high temporal density, allowing the model to capture both local and global features of the signal simultaneously;
(4) Window Size 2048, Stride 80: This is the segmentation method with the largest time span and the lowest temporal density. Each image contains 2048 data points, with an overlap of 96.1% between adjacent images.

**Figure 4:**
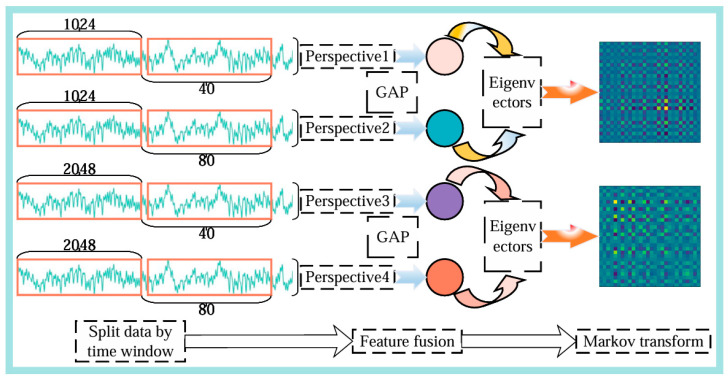
Schematic diagram of multi-view joint optimization for feature extraction.

As shown in Figure 4, the data segmented using method (1) and method (2) (which generates images that are less dense along the time axis but contain more temporal information) are fused to form Feature Vector 1. Similarly, the data segmented using method (3) and method (4) (which have the largest time span and the lowest temporal density) are fused to form Feature Vector 2. After obtaining Feature Vector 1 and Feature Vector 2, a Markov transformation is applied to convert the feature vectors into two-dimensional images, better adapting them to the input requirements of subsequent neural networks. By introducing sliding window mechanisms or adaptive feature selection, the model can dynamically adjust and analyze signals under varying load and environmental conditions. Multi-view joint optimization of feature extraction partitions and feature fusion yields image sequences with different time scales and densities. This approach provides more comprehensive and rich feature information for subsequent feature extraction and model training.

### 3.2. Improved Capsule Network Architecture

#### 3.2.1. Internal Structure of Capsule Network

**Figure 5:**
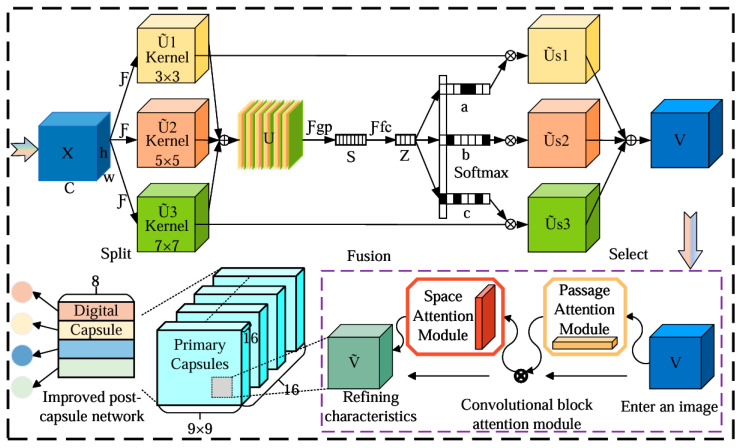
Schematic diagram of the improved capsule network workflow. X is the input SKNet data; Ũ1, Ũ2, Ũ3 are SKNet channels with convolved nuclei of different sizes; Ũs1, Ũs2, Ũs3 are three different channels after feature extraction by SKNet; V is the final output data of SKNet and also the input data of CBAM attention mechanism. Ṽ is the data processed by CBAM, and all arrows show the data direction.

The core of the capsule network lies in its unique “capsule” units, which not only represent the existence of entities but also their pose, size, orientation, and other attribute information. As shown in Figure 5, a network architecture is designed that includes multiple convolutional layers, SKNet, capsule layers, and the CBAM attention mechanism [25]. The convolutional layers are used to initially extract feature maps from the vibration signals. SKNet dynamically guides the feature maps into channels with different-sized convolutional kernels for further feature extraction. The CBAM attention mechanism introduces two modules—channel attention and spatial attention—which enhance the network’s focus on the input data. Particularly in complex visual scenes, CBAM helps the model more accurately locate and process important feature information, thereby improving overall performance. The capsule layers further transform these feature maps into capsule representations and use the dynamic routing algorithm to achieve effective information transmission and integration. The primary code for the improved capsule network model proposed in this paper is presented in Appendix A.

#### 3.2.2. Capsule Network Parameters

To optimize the performance of the capsule network, detailed adjustments were made to various parameters, including the size and number of convolutional kernels, the dimension and number of capsules, and the number of iterations in the dynamic routing process. Through experimental validation, a set of capsule network parameter settings suitable for the transfer task on imbalanced bearing fault datasets was determined, as shown in Table 2.

#### 3.2.3. Output Shape and Parameter Calculation of the Siamese Capsule Network

In this study, the Siamese capsule network consists of two capsule networks with identical architectures that share weights. This means that both sub-networks use the same parameters when performing similarity judgments. The weight-sharing approach effectively reduces the number of parameters that need to be independently trained, thereby decreasing computational overhead.

The reduction in parameter quantity primarily stems from the shared weights between sub-networks, ensuring that each sub-network has the same parameter count. Due to the shared parameters, during training, the model only needs to optimize a single set of common weights rather than multiple independent sets, as shown in Table 3.

### 3.3. Transfer Network Construction and Workflow

The core of the Siamese neural network lies in its unique architecture, which utilizes two identical neural network branches with shared weights to simultaneously process a pair of input samples and output their feature representations. These feature representations are subsequently used to compute the similarity or distance between the input samples. To achieve model transfer, one of the key strategies of the Siamese neural network is to leverage pre-trained weight information from the source domain.

The network is pre-trained using source domain data, where the two branches of the Siamese neural network are trained on a sufficient number of source domain samples to determine their shared weight parameters. This ensures that the network can fully learn the intrinsic patterns and features of the source domain data.

To retain useful features from the source domain, the weights of the network layers responsible for extracting low-level features are frozen.

Above the frozen pre-trained layers, new fully connected layers are added. The weights of these new layers are initialized and need to be fine-tuned using a small number of samples from the target domain. The purpose of fine-tuning is to enable the new layers to adapt to the data distribution of the target domain and optimize the performance of the Siamese neural network on the target domain.

After fine-tuning, the Siamese neural network can accept a pair of input samples from the target domain and output their feature representations. These feature representations are then used to compute the similarity or distance between the input samples, thus enabling transfer diagnosis of bearing faults.

Through these steps, an efficient and effective Siamese neural network model for bearing fault transfer diagnosis has been constructed. This model not only leverages knowledge from the source domain but also enhances its performance on the target domain through transfer learning and fine-tuning strategies.

**Figure 6:**
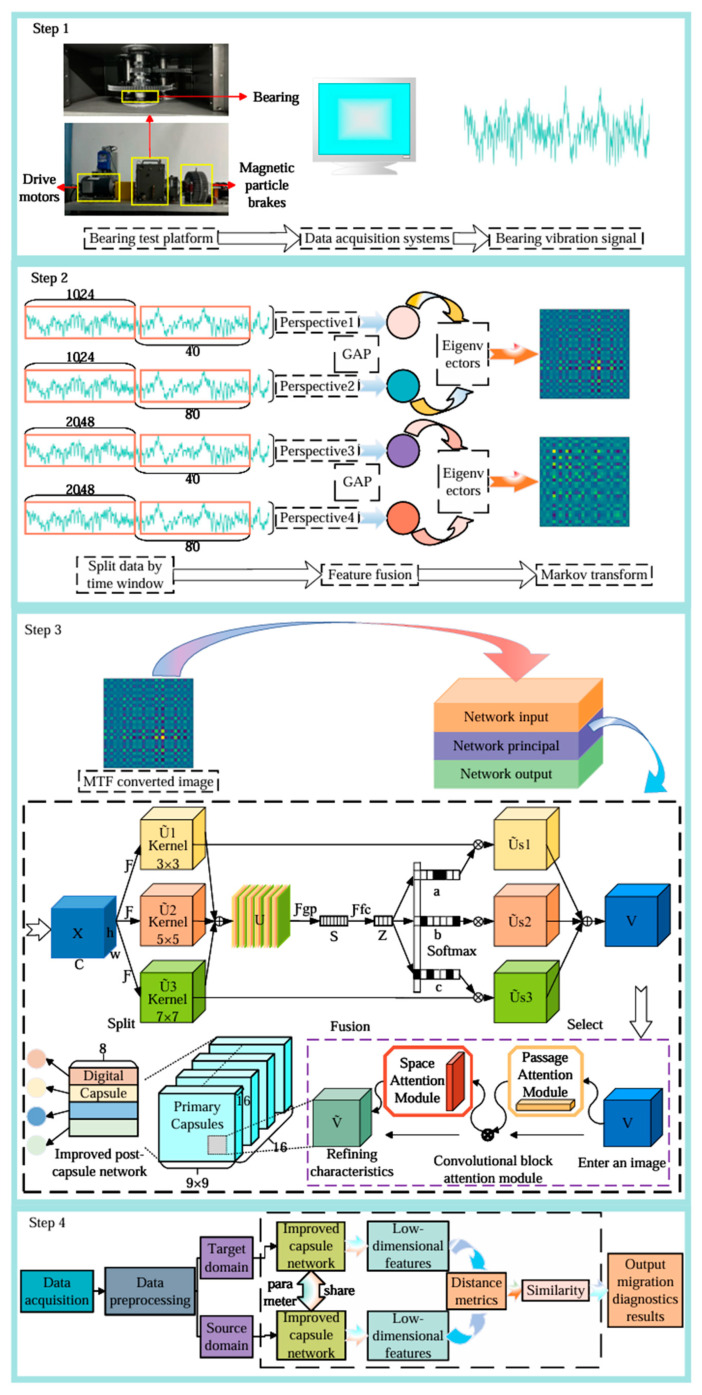
Schematic diagram of the transfer network workflow.

The proposed model converts vibration signals into Markov transformation [26] images, which are used as inputs. An improved Siamese capsule network is employed as the fault diagnosis model to perform feature extraction and similarity comparison on the input images. The workflow of the proposed method is illustrated in Figure 6.

(1) Source Domain Data Preparation: Traditional rolling bearing vibration signals serve as the source domain data. Following the preprocessing steps outlined in Section 3.1, the corresponding source domain dataset is constructed;
(2) Model Training on Source Domain: The fault diagnosis model based on the Siamese capsule network is trained using the constructed source domain dataset. The Siamese capsule network comprises two identical branches with shared weights, enabling it to learn the spatial hierarchical structure and dynamic routing relationships between features. Ensure that the model is fully trained to obtain a well-performing source domain fault diagnosis model;
(3) Target Domain Data Preparation: Another portion of the rolling bearing vibration signals is selected as target domain data. Following the same preprocessing steps outlined in Section 3.1, the corresponding target domain dataset is constructed;
(4) Parameter Transfer: The architecture and weight parameters of the capsule network branches from the source domain fault diagnosis model are transferred to the target domain diagnosis model. According to the predefined parameter update strategy, certain transferred parameters (typically the capsule layers responsible for extracting low-level features) are retained and frozen to ensure they do not participate in subsequent training. This step ensures the transfer and retention of knowledge;
(5) Fine-Tuning on Target Domain: Using the target domain training dataset, the target domain diagnosis model is trained based on the frozen parameters. The remaining network parameters and weights are fine-tuned. The goal of fine-tuning is to enable the newly added capsule layers to adapt to the data distribution of the target domain and optimize the performance of the Siamese capsule network on the target domain;
(6) Model Testing: After training the target domain diagnosis model, it is tested using the test dataset. The diagnostic accuracy of the model for bearing faults is evaluated by computing the similarity or distance between test sample pairs. The final diagnostic accuracy is used to measure the model’s performance.

## 4. Experimental Verification

This paper validates the effectiveness of the proposed model for fault diagnosis in imbalanced bearing datasets using two different datasets. To align with real-world industrial applications, the applicability of the model under high-noise conditions is also discussed, and the results are presented using visualization charts. The experiments were conducted on a Windows 11 system with an i5-13600KF processor and an RTX3060Ti 32 GB GPU (ASUS, Shenzhen, China), using the PyCharm platform (v2022.1.4), Python programming language, and the PyTorch deep learning framework.

### 4.1. Data Augmentation

After performing multi-view data segmentation as described in Section 3.1, the data were standardized and normalized. Image augmentation strategies, including 20% horizontal flipping, 20% vertical flipping, and 10% clockwise rotation by 90 degrees, were applied to significantly enhance the diversity and complexity of the dataset. Images were normalized to ensure consistency in the input data. However, the target domain dataset was not augmented, with the aim of testing the model’s diagnostic capability on target domain datasets with fewer fault samples.

### 4.2. CWRU Bearing Dataset

The rolling bearing dataset used in this experiment is sourced from the Case Western Reserve University (CWRU) Bearing Data Center [27]. Accelerometer sensors were used to collect the vibration acceleration signals of the bearings, which were installed near the motor-driven end bearing housing. The sampling frequency was 12 kHz. Based on the location of the fault, the bearing conditions are categorized into four types: roller element fault, inner race fault, outer race fault, and normal operation. Faulty bearings were installed on the test motor, and the motor was operated under different loads to record vibration data at various motor load levels. The fault signals collected in this study were obtained from the deep groove ball bearing SKF6205 under three different operating conditions, with motor loads ranging from 1 to 3 HP. The signals were collected as acceleration data with a sampling frequency of 12 kHz. The fault mode of the bearing is pitting.

In the experiment, 10 types of data were selected. To simulate the practical engineering scenario where it is sometimes difficult to collect rich fault data under multiple operating conditions, the experiment only collected bearing fault signals under one operating condition (speed of 1797 rpm) for training. The dataset includes normal data and three different fault types with three different sizes (in inches), labeled from 0 to 9. The dataset is divided into training data and test data, with 100 samples in the training set and 900 samples in the test set. The dataset is detailed in Table 4.

#### 4.2.1. Comparative Ablation Study (CWRU Dataset)

**Figure 7:**
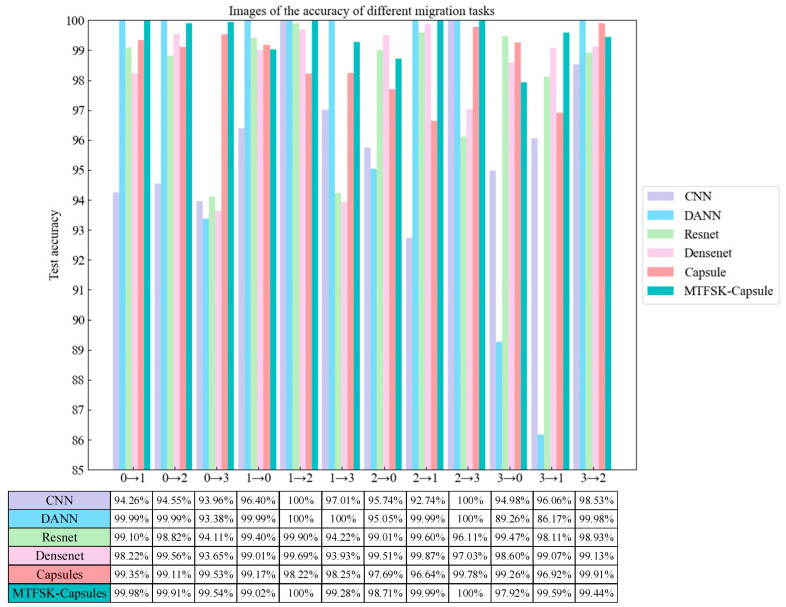
Comparative ablation accuracy for different transfer tasks (CWRU dataset).

The results of the comparative ablation study are shown in Figure 7. After 50 iterations, the proposed model achieved an average accuracy of 99.48% across different transfer tasks. The experimental results demonstrate that the data preprocessing method proposed in this paper, which generates image sequences with different time scales and densities, not only retains important information from the original signals but also enhances the readability and interpretability of the data, thereby improving the model’s accuracy. Compared to five other models, the proposed method uses SKNet to capture information between feature channels, enhancing the model’s ability to utilize useful features.

#### 4.2.2. Noise Resistance Experiment (CWRU Dataset)

**Figure 8:**
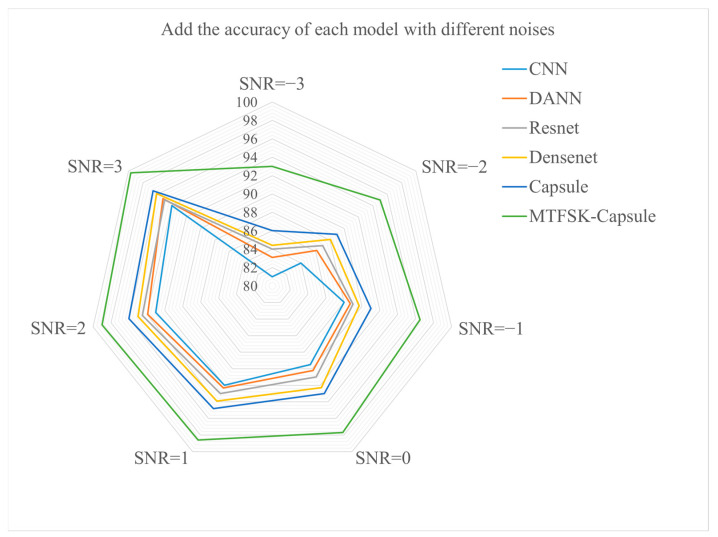
Accuracy of different models under various signal-to-noise ratios (CWRU dataset).

To better reflect real-world scenarios, Gaussian white noise with different signal-to-noise ratios (SNRs) was added to the test set. Figure 8 intuitively demonstrates the precision performance of the model under SNR conditions of −3, −2, −1, 0, 1, 2, and 3 dB. The observations show that the proposed model exhibits excellent fault detection performance even in environments with significant noise interference. In the extreme −3 dB SNR environment, the accuracy remains as high as 94.6%. Compared to traditional neural networks, the capsule network enhances its noise resistance capabilities by introducing more complex and structured data representation and processing methods, such as innovative “capsule” structures, unique feature vector representations, and dynamic routing strategies. This paper first applies the Markov transformation to convert bearing fault data into a two-dimensional image format, where the noise in the data primarily manifests as changes in image pose, relational information, occlusions, and deformations. Additionally, the time series of the data can be incorporated, which helps the capsule network more effectively handle these noise interferences. This transformation further validates the proposed model’s ability to extract and detect fault features under extreme conditions.

#### 4.2.3. Dimensionality Reduction Visualization (CWRU Dataset)

**Figure 9:**
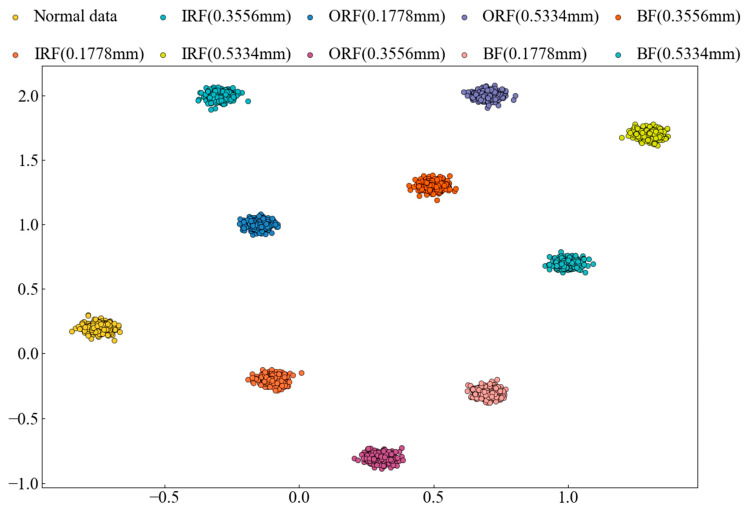
Dimensionality reduction visualization of the model on CWRU transfer task 0→1 (CWRU dataset).

t-SNE is a nonlinear dimensionality reduction method that transforms high-dimensional data into a two-dimensional or three-dimensional visual representation. In neural networks, t-SNE can be used to analyze the feature representations of intermediate layers, mainly in the following two aspects:

(1) Class Boundary Identification: t-SNE visualization allows for the observation of distinct separations between different data classes, providing insights into how the network learns and distinguishes class information;
(2) Clustering Structure in Feature Space: t-SNE highlights the clustering of similar samples within the feature space, helping to understand the feature representation in the intermediate layers and the network’s classification process.

t-SNE is used to map the feature information from the network’s output layer onto a two-dimensional plane. Figure 9 presents the results of the model for the CWRU transfer task 0→1, clearly showing the distinct separation between each fault state.

#### 4.2.4. Confusion Matrix (CWRU Dataset)

**Figure 10:**
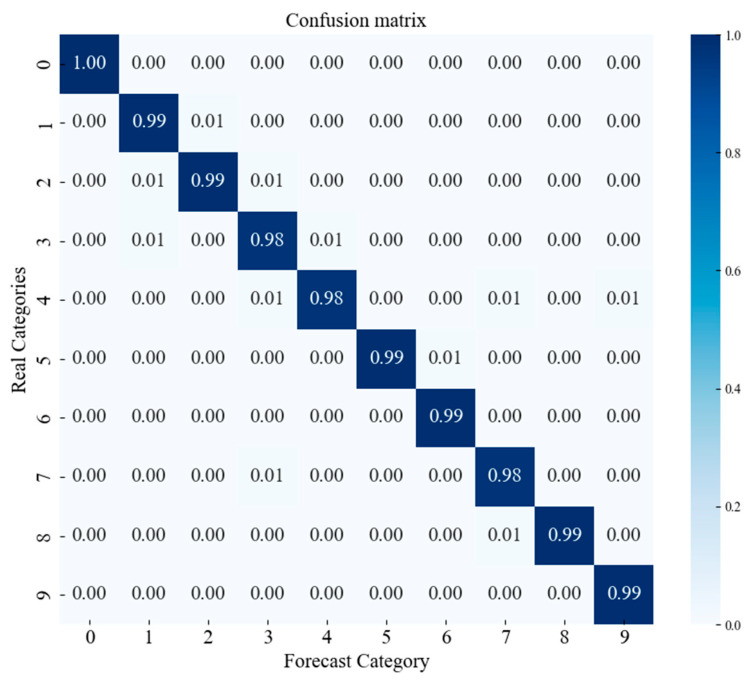
Confusion matrix of the model (CWRU dataset).

The confusion matrix is a tool for evaluating and analyzing model performance, providing a clear view of the detailed classification outcomes and the accuracy of the model for different categories of data. As shown in Figure 10, the confusion matrix is used to comprehensively assess the model’s performance on the following label classification tasks: Label 0 represents normal data, labels 1–3 represent inner race fault data, labels 4–6 represent outer race fault data, and labels 7–9 represent roller element fault data.

### 4.3. Laboratory Bearing Dataset

**Figure 11:**
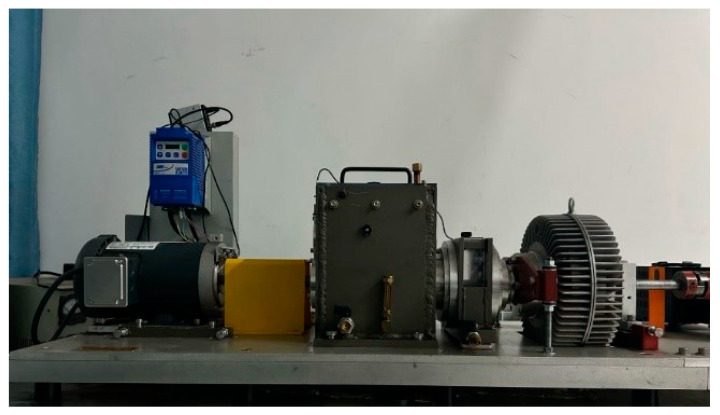
Laboratory bearing fault test rig.

**Figure 12:**
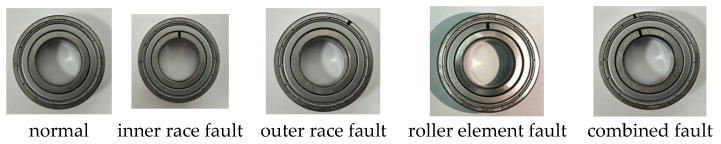
Various fault categories of bearings.

Figure 11 shows the laboratory bearing fault test rig. The experimental dataset was collected at a sampling frequency of 25.6 kHz and includes vibration signals from five different health states: inner race fault, outer race fault, roller element fault, combined inner and outer race faults, and normal conditions. The different fault locations on the bearing are shown in Figure 12. The laboratory rolling bearing data partitioning is shown in Table 5.

#### 4.3.1. Comparative Ablation Study (Laboratory Bearing Dataset)

**Figure 13:**
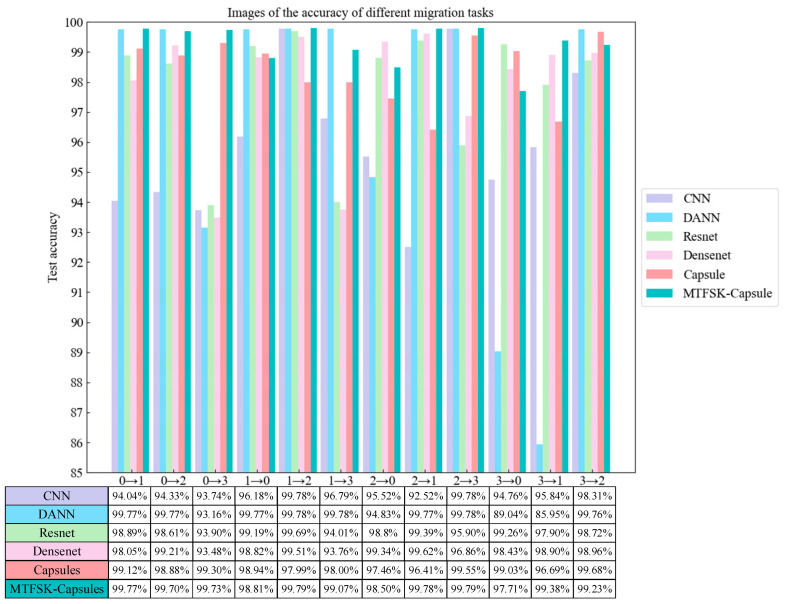
Comparative ablation accuracy for different transfer tasks (laboratory bearing dataset).

As shown in Figure 13, after 50 iterations, the model achieved an average accuracy of 99.27% on different transfer tasks using the laboratory-bearing fault dataset. The experimental results demonstrate that the model performs excellently across different datasets, proving its strong generalization capability.

#### 4.3.2. Noise Resistance Experiment (Laboratory Bearing Dataset)

**Figure 14:**
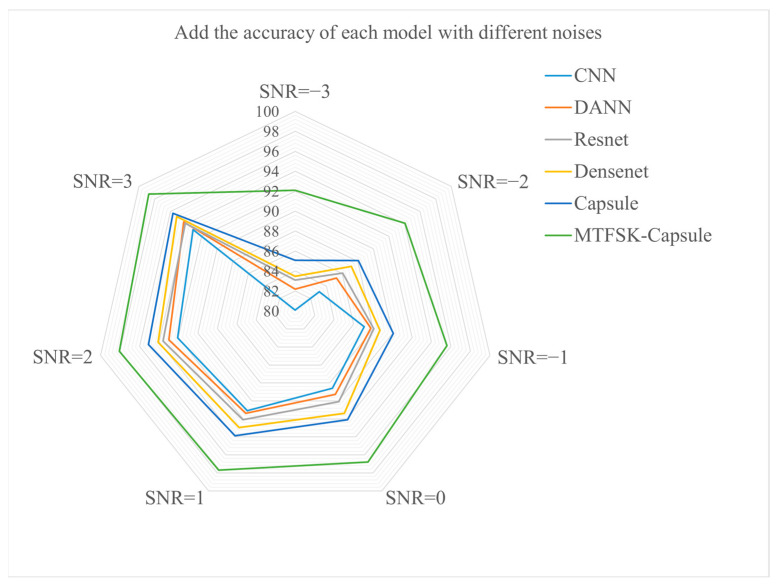
Accuracy of different models under various signal-to-noise ratios (laboratory bearing dataset).

The same method was used to test the model’s noise resistance on the laboratory bearing fault dataset, where Gaussian white noise with signal-to-noise ratios (SNRs) of −3, −2, −1, 0, 1, 2, and 3 dB was added to the test set. As shown in Figure 14, the proposed model performed well on the laboratory-bearing fault dataset, demonstrating its excellent generalization and robustness.

#### 4.3.3. Dimensionality Reduction Visualization (Laboratory Bearing Dataset)

**Figure 15:**
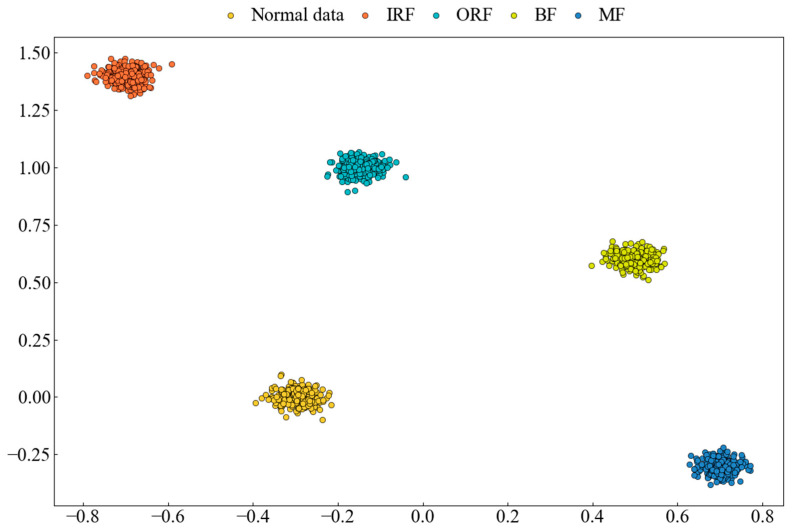
Dimensionality reduction visualization of the model on transfer task A→B (laboratory bearing dataset).

Using t-SNE, the feature information from the network’s output layer is transformed and visualized on a two-dimensional plane. Figure 15 shows the results of the model on the transfer task A→B using the laboratory bearing fault dataset, clearly demonstrating good separation for each fault state.

#### 4.3.4. Confusion Matrix (Laboratory Bearing Dataset)

**Figure 16:**
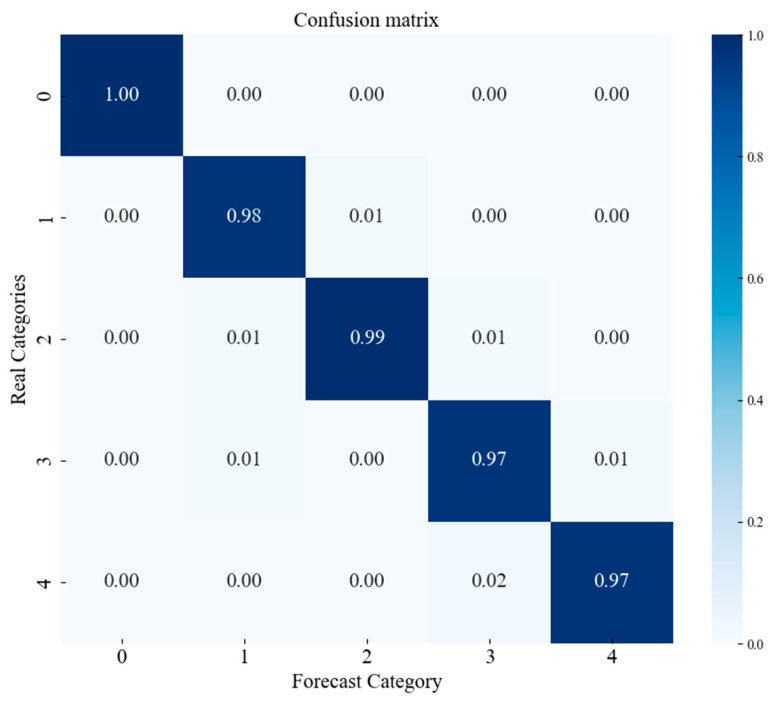
Confusion matrix of the model (laboratory bearing dataset).

The confusion matrix is a tool for evaluating and analyzing model performance, providing a clear view of the detailed classification outcomes and the accuracy of the model for different categories of data. As shown in Figure 16, the confusion matrix is used to comprehensively assess the model’s performance on the following label classification tasks: Label 0 represents normal data, labels 1–3 represent inner race fault data, labels 4–6 represent outer race fault data, and labels 7–9 represent roller element fault data.

## 5. Conclusions

This paper addresses the challenges of small-sample bearing fault diagnosis, including limited sample availability, high noise interference, difficulty in feature extraction, and long transfer learning times, by proposing a method that combines an improved capsule network with a Siamese neural network. Through multi-view data partitioning and Markov transformation, the diversity of the data is effectively enriched, and the visualization features of the signals are enhanced. By leveraging the dynamic routing mechanism of the capsule network, fault features are captured and integrated, significantly improving the model’s feature representation and robustness. The introduction of the Siamese network for shared weight optimization in feature matching, combined with SKNet for dynamic feature fusion, enhances transfer efficiency, reduces the number of parameters, and achieves lightweight model processing, effectively reducing model complexity and shortening transfer time. Experimental results demonstrate that this method can accurately identify faults in environments with limited samples and high noise, significantly improving diagnostic accuracy and drastically reducing transfer learning time. Although the model demonstrates strong generalization capabilities on two datasets, its performance still requires improvement in certain specialized tasks or conditions. Despite using both a public dataset and a private dataset in this study, the diversity of the datasets remains somewhat limited and may not fully represent real-world mechanical fault diagnosis scenarios. Additionally, experiments were conducted under stable operating conditions, whereas real-world industrial environments are continuously changing. Therefore, the proposed model needs further testing in practical environments.

Future Research Plans: (1) Conduct further validation and research using more complex and diverse actual industrial fault data. (2) While Markov transformation preserves local dynamic characteristics, its static representation limits its ability to capture long-term fault evolution. Future research will explore the integration of dynamic sequence models to complement the existing framework and enhance the model’s capability to handle long-term dynamic changes.

## Figures and Tables

**Table 1:** Steps and explanations for calculating Euclidean distance.

Step	Operation	Formula/Detailed Description
1	Determine Input Vectors	Assume there are two n-dimensional vectors, X and Y, represented as X = (x1, x2, …, xn) and Y = (y1, y2, …, yn).
2	Calculate Differences in Each Dimension	For each dimension i (i = 1, 2, …, n), calculate the difference between the vectors X and Y in that dimension, i.e., di = xi−yi. This will generate a difference vector D = (d1, d2, …, dn).
3	Compute Squares of the Differences	For each element di (i = 1, 2, …, n) in the difference vector D, compute its square value, i.e., di^2^ = (xi − yi)^2^. This will generate a squared difference vector D^2^ = (d1^2^, d2^2^, …, dn^2^).
4	Calculate the Sum of Squares	Sum all the elements in the squared difference vector D^2^ to obtain the sum of squares S, i.e., S = ∑(di^2^) = d1^2^ + d2^2^ + … + dn^2^. This is the total sum of the squares of the differences between the two vectors.
5	Compute Euclidean Distance	Take the square root of the sum of squares S to obtain the Euclidean distance d, i.e., $d=\sqrt{S}=\sqrt{\left({d1}^{2}+{d2}^{2}+...+{dn}^{2}\right)}$

**Table 2:** Improved capsule network parameters.

Layer	Network Parameters	
MTF		
Conv (SKNet)	(64,64), K = 3, S = 1, P = 1	SKNet Channel 1
	(64,64), K = 5, S = 1, P = 2	SKNet Channel 2
	(64,64), K = 7, S = 1, P = 3	SKNet Channel 3
Primary capsule	Num_conv_units = 16	Primary capsule
	In_channels = 64	Input
	Out_channels = 16	Output Channels
	Kernel_size = 9	Kernel Size
	Stride = 2	Stride
Digit_caps	In_dim = 16	Input Capsule Dimension
	In_caps = 1296	Input Capsule Number
	Out_caps = 4	Output Capsule Number
	Out_dim = 8	Output Capsule Vector Dimension
	Num_routing = 16	Routing Algorithm Iterations

**Table 3:** Output shape and computational parameters of the Siamese capsule network.

Module	Output Shape	Computational Parameters
Conv2d	(64, 26, 26)	2368
ReLU	(64, 26, 26)	0
SKConv	(64, 26, 26)	77,056
PrimaryCaps	(16, 6, 6)	200,704
Reconstruction (decoder)	(28 × 28)	136,960
Reshape	(28, 28)	0
Flatten	(784)	0
Fully Connected	(num_classes)	0
Softmax	(num_classes)	0

**Table 4:** CWRU rolling bearing data.

Label	Bearing Condition	Damage Location	Rotation Speed	Damage Length
0	Normal	Normal	1797 rpm	0
1	Fault	Inner Race	1797 rpm	0.007
2	Fault	Inner Race	1797 rpm	0.014
3	Fault	Inner Race	1797 rpm	0.021
4	Fault	Outer Race	1797 rpm	0.007
5	Fault	Outer Race	1797 rpm	0.014
6	Fault	Outer Race	1797 rpm	0.021
7	Fault	Roller Element	1797 rpm	0.007
8	Fault	Roller Element	1797 rpm	0.014
9	Fault	Roller Element	1797 rpm	0.021

**Table 5:** Laboratory rolling bearing data.

Dataset	Category	Sample Count	Fault Location
A	training set	800	normal
	test set	200	
B	training set	100	inner race
	test set	900	
C	training set	100	outer race
	test set	900	
D	training set	100	roller element
	test set	900	
E	training set	100	combined fault
	test set	900	

## Data Availability

The CWRU Bearing Dataset is available for open access at https://engineering.case.edu/bearingdatacenter (accessed on 1 November 2024). The Laboratory Bearing Dataset was collected by the author’s institution and is not available for public use.

## Appendix A

Below is the main network of the model.

import torch.nn as nn import torch from .baseModels import PrimaryCaps, DigitCaps from cbam import cbam_block from SKNet import SKConv import torch.nn.functional as F device = “cuda” if torch.cuda.is_available() else “cpu” class CapsNet(nn.Module): ‘‘‘ Basic implementation of capsule network layer ‘‘‘ def __init__(self, img_w, img_h, num_classes): super(CapsNet, self).__init__() self.num_classes = num_classes # Conv2d layer self.conv1 = nn.Conv2d(1, 64, 3, 1) self.relu = nn.ReLU(inplace = False) # self.cbam = cbam_block(64) self.sknet = SKConv(64, M = 3, G = 1, r = 3, outFeatures = 64) # Primary capsule self.primary_caps = PrimaryCaps(num_conv_units = 16, in_channels = 64, out_channels = 16, kernel_size = 9, stride = 2) self.layerNorm = nn.LayerNorm # Digit capsule self.digit_caps = DigitCaps(in_dim = 16, in_caps = 1296, out_caps = num_classes, out_dim = 8, num_routing = 16) self.digit_caps.setDevice(device) # Reconstruction layer self.decoder = nn.Sequential( nn.Linear(num_classes * 8, 128), nn.ReLU(inplace = True), nn.Linear(128, 256), nn.ReLU(inplace = True), nn.Linear(256, img_w*img_h), # nn.Sigmoid() ) def forward(self, x): out1 = self.conv1(x) # print(0, out1.shape) out2 = self.relu(out1) out3 = self.sknet(out2) # print(1, out3.shape) out4 = self.relu(out3) # out = F.dropout(out, 0.5) out5 = self.primary_caps(out4) # print(2, out5.shape) out6 = self.digit_caps(out5) # print(3, out6.shape) # Shape of logits: [batch_size, out_capsules] logits = torch.norm(out6, dim=–1) pred = torch.eye(self.num_classes).to(device).index_select(dim = 0, index = torch.argmax(logits, dim = 1)) # Reconstruction batch_size = out6.shape[0] reconstruction = self.decoder((out6 * pred.unsqueeze(2)).contiguous().view(batch_size, –1)) return logits, reconstruction from torch import nn import torch class Exping(nn.Module): def __init__(self): super(Exping, self).__init__() def forward(self, x): return torch.exp(x) * torch.sin(x) # Apply Exping to the corresponding layers in the neural network. model = nn.Sequential( nn.Linear(10, 20), Exping(), nn.Linear(20, 1) ) class SKConv(nn.Module): def __init__(self, features, M, G, r, stride = 1, L = 16, outFeatures = 128): super(SKConv, self).__init__() d = max(int(features / r), L) self.M = int(M) self.features = features self.outFeatures = outFeatures self.convs = nn.ModuleList([]) for i in range(self.M): # Convolutions with different kernel sizes self.convs.append( nn.Sequential( nn.Conv2d(features, self.outFeatures, # kernel_size = 3 + i * 2, kernel_size = (1, 3 + i * 2), stride = stride, padding = (0, i+1), groups = G), nn.BatchNorm2d(self.outFeatures), nn.ReLU(inplace = False))) self.fc = nn.Linear(self.outFeatures, d) self.fcs = nn.ModuleList([]) for i in range(self.M): self.fcs.append(nn.Linear(d, outFeatures)) self.softmax = nn.Softmax(dim = 1) def forward(self, x): for i, conv in enumerate(self.convs): fea = conv(x).unsqueeze(dim = 1) if i == 0: feas = fea else: feas = torch.cat([feas, fea], dim = 1) fea_U = torch.sum(feas, dim = 1) fea_s = fea_U.mean(–1).mean(–1) fea_z = self.fc(fea_s) for i, fc in enumerate(self.fcs): # print(i, fea_z.shape) vector = fc(fea_z).unsqueeze(dim = 1) # print(i, vector.shape) if i == 0: attention_vectors = vector else: attention_vectors = torch.cat([attention_vectors, vector], dim = 1) attention_vectors = self.softmax(attention_vectors) attention_vectors = attention_vectors.unsqueeze(–1).unsqueeze(–1) fea_v = (feas * attention_vectors).sum(dim = 1) return fea_v
